# First Report of *Angiostrongylus cantonensis* (Nematoda: Angiostrongylidae) Infections in Invasive Rodents from Five Islands of the Ogasawara Archipelago, Japan

**DOI:** 10.1371/journal.pone.0070729

**Published:** 2013-08-07

**Authors:** Toshihiro Tokiwa, Takuma Hashimoto, Tatsuo Yabe, Noriyuki Komatsu, Nobuaki Akao, Nobuo Ohta

**Affiliations:** 1 Section of Environmental Parasitology, Graduate School of Tokyo Medical and Dental University, Tokyo, Japan; 2 Japan Wildlife Research Center, Tokyo, Japan; 3 Rat Control Consulting, Kanagawa, Japan; 4 Division of Research and Development, Civil International Corporation, Tokyo, Japan; University of Minnesota, United States of America

## Abstract

**Background:**

*Angiostrongylus cantonensis* (Chen, 1935) is a parasite of murid rodents and causative agent of human neuro-angiostrongyliasis. In 2011, the Ogasawara Islands in the western North Pacific were assigned a World Natural Heritage site status. The occurrence of *A. cantonensis* is well documented in the Chichijima, Hahajima, and Anijima Islands. However, the occurrence of *A. cantonensis* in the other islands of the Ogasawara Islands has not been reported.

**Methodology/Principal Findings:**

Between March 2010 and July 2011, 57 *Rattus norvegicus* and 79 *R. rattus* were collected from 9 islands (the Hahajima group: Anejima, Imoutojima, Meijima, Mukohjima, and Hirajima; Chichijima group: Minamijima; Mukojima group: Nakoudojima and Yomejima; and Iwojima group: Iwojima). Adult nematodes were found in the pulmonary artery of 46 *R. norvegicus* collected in the 5 islands of the Hahajima group (Anejima, Meijima, Imoutojima, Hrajima, and Mukohjima Islands). These nematodes were identified by molecular analysis as *A. cantonensis.* Comparison of the mitochondrial DNA sequences confirmed that all the samples from the Ogasawara Islands shared only a single lineage of *A. cantonensis*, which has been previously detected in the Okinawa, Hawaii, and Brazil.

**Conclusions/Significance:**

We describe new endemic foci of rat angiostrongyliasis in the Hahajima group (Anejima, Meijima, Imoutojima, Hirajima, and Mukohjima Islands) of the Ogasawara Islands. These findings indicate that the endemic foci of *A. cantonensis* are widely distributed in the Ogasawara Islands. Although human cases have not yet been reported in the Ogasawara Islands, the widespread detection of *A. cantonensis* could be of importance from the perspective of public health.

## Introduction

Neuro-angiostrongyliasis due to infection with *Angiostrongylus cantonensis* (Chen, 1935) is an important public health concern in many countries [1], [2]. *A. cantonensis* is widespread in the tropical and subtropical areas. It is reported mainly in Asia, the Pacific Islands, the Caribbean Islands, Australia, and the USA [3], [4]. However, recent studies have suggested an increasing incidence, broader distribution [5], and sudden appearance in areas previously believed to be free from infections with *A. cantonensis* [6], [7], [8], [9].

The Ogasawara Islands is group of more than 30 small subtropical islands in the western North Pacific, located approximately 1,000 km south of mainland Japan. The Ogasawara Islands extend approximately 400 km from north to south and consist of the Ogasawara archipelago (the Chichijima, Hahajima, and Mukojima groups), the Iwojima groups, and isolated islands in the surrounding area. The Ogasawara Islands consists of only two of inhabited Islands of Chichijima (the biggest island in the Chichijima group) and Hahajima (the biggest island in the Hahajima group) Islands. In 2011, the Ogasawara Islands was assigned World Natural Heritage site status. It is an area known to be endemic for *A. cantonensis.* The occurrence of *A. cantonensis* is well documented in the Chichijima [10], [11], [12], [13], [14], [15], Hahajima [15], [16], [17], and Anijima (belonging to Chichijima group) Islands [18]. However, the occurrence of *A. cantonensis* in the other islands of the Ogasawara Islands has not been reported.

Nuclear small subunit (SSU) rRNA sequences generally show little variation within a nematode species, but substantial divergence among species, allowing for species differentiation [9], [19], [20]. On the other hand, the mitochondrial cytochrome *c* oxidase subunit I (*coxI*) gene has proved to be a powerful marker in resolving phylogenetic relationships within closely related *Angiostrongylus* species [20], [21], and has provided further information to increase understanding of the genetic differences among *A. cantonensis* isolates [20], [22]. In a previous study, we showed the presence of 7 distinct haplotypes (ac1, ac2, ac3, ac4, ac5, ac6, and ac7) of the *A. cantonensis coxI* gene in Asia [20]. Subsequently, Monte et al. (2012) [22] found the presence of 3 distinct haplotypes (ac5, ac8, and ac9) in Brazil. These results supports that the appearance of *A. cantonensis* in these regions is likely a results of multiple introduction of infected hosts via human related transportation [20], [22].

In the present study, we conducted extensive research on the presence of *A. cantonensis* among invasive rats in the Ogasawara Islands. In addition, we have genotyped and investigated genetic differences among *A. cantonensis* on these islands by using SSU rRNA and *coxI* genes for monitoring the spread of *A. cantonensis* lineages.

## Materials and Methods

### Sampling

**Figure 1:**
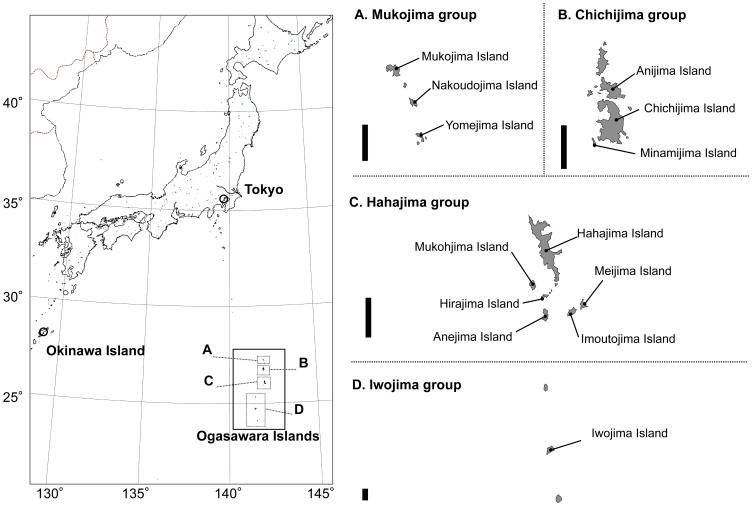
Map showing the location of the Ogasawara Islands and the four islands group. **Bar indicated 5 km long.**

Between March 2010 and July 2011, a total number of 136 rats (57 Norway rats, *R. norvegicus,* and 79 Black rats, *R. rattus*) were collected from 9 islands (the Hahajima group: Anejima, Imoutojima, Meijima, Mukohjima, and Hirajima; Chichijima group: Minamijima; Mukojima group: Nakoudojima and Yomejima; and Iwojima group: Iwojima) in the Ogasawara Islands (Fig. 1) by using cages and snap traps. Animals were taxonomically classified according to morphological criteria (weight, length of head and body, length of tail, length of the ear, color etc.), euthanized with CO_2_ and dissected in the field. Lung and heart samples were fixed with 70% ethanol. The *Angiostrongylus* worms in the plumonary arteries of these rats were collected by macroscopic inspection. *A. cantonensis* collected from Hawaii (Alicata strain) were kindly provided Drs. N. Nonaka and T. Irie (Miyazaki Univ., Japan). These worms were preserved at −30°C until DNA extraction. Detailed information about the host, prevalence, and sampling date is included in Table 1.

**Table 1:** Results of survey of *Angiostrongylus* worms among rodents in the Ogasawara Islands.

Island		Host species	No. of examined	No. of positive	Prevalence (%)	Data of captured
Mukojima group	Nakoudojima	*Rattus rattus*	12	0	0.0	Jul, 2011
	Yomejima	*R. rattus*	35	0	0.0	Jul, 2011
Chichijima group	Minamijima	*R. rattus*	32	0	0.0	Feb, 2011, Dec, 2010
Hahajima group	Anejima	*R. norvegicus*	2	2	100.0	Oct, 2010
	Hirajima	*R. norvegicus*	5	3	60.0	Oct, 2010
	Imoutojima	*R. norvegicus*	22	21	95.5	Oct, 2010
	Meijima	*R. norvegicus*	17	16	94.1	Oct, 2010
	Mukohjima	*R. norvegicus*	5	4	80.0	Oct, 2010
Iwojima group	Iwojima	*R. norvegicus*	6	0	0.0	Mar, 2010, Aug, 2010
Over all prevalence			136	46	33.8	

### Ethical Statement

Sample collecting in the Ogasawara Islands by TH was carried out under the permission of the Ministry of the Environment, Japan. An independent Animal Ethics Committee of the Tokyo Medical and Dental University approved these studies (permit number 100170), in accordance with the Act on Welfare and Management of Animals of Japanese government.

### DNA Extraction, PCR Amplification, and Sequencing

The SSU rRNA gene isolated from the worms collected from 5 islands in the Hahajima group was amplified and sequenced. The mitochondrial *coxI* gene was amplified and sequenced from 46 individuals of Ogasawara isolates and two Hawaiian isolates of *A. cantonensis*. Genomic DNA was extracted using sodium hydroxide (NaOH) direct lysis protocol [23]. SSU rRNA and *coxI* genes were amplified by PCR using SSU (SSU18A, 5′-AAAGTTAAGCCATGCATG-3′; SSU26R, 5′-CATTCTTGGCAAATGCTTTCG-3′) [24] and *cox1* (cox1F, 5′-TTTGTTTTGATTTTTTGGTC-3′; cox1R, 5′-AGGATAAATCTAAATACTTACGAGGA-3′) primers [20], respectively. The amplification conditions consisted of an initial denaturation at 94°C for 2 min; followed by 35 cycles of 94°C for 30 s, 60°C for 30 s, and 72°C for 30 sec; followed by a final extension step at 72°C for 2 min. Each 20-µl reaction mixture consisted of 14.8 µl of water, 2 µl of 10× PCR Buffer (Bioneer Co., Korea), 1.6 µl of dNTPs (10 mM), 0.2 µl of each primer (1.6 µM), 0.1 µl of *Top* DNA polymerase (0.5 U/µM) (Bioneer Co., Korea), and 1 µl of sample DNA. The PCR reactions were analyzed by electrophoresis on a 1.5% agarose gel and visualized by UV illumination by ethidium bromide staining. The PCR products were purified by Exosap-IT (GE Healthcare, England). Sequencing was performed using the BigDye Terminator version 3.1 Cycle Sequencing kit (Applied Biosystems, USA) according to the manufacturer’s instruction, and determined by ABI3100 automatic sequencer (Applied Biosystems, USA). All products were sequenced in both directions. Sequences were visualized by chromatograms, and then, manually corrected using Finch TV software (Geospiza, USA).

### Sequence Analysis

A BLAST search (National Center of Biotechnology Information public databases; http://blast.ncbi.nlm.nih.gov/Blast.cgi) was carried out to elucidate any similarities between the obtained SSU rRNA and *coxI* sequences and the previously published sequences. Multiple sequence alignment was carried out using the online version of MAFFT ver 6.0 [25] with the option Q-INS-I [26].

## Results

### Prevalence of *A. cantonensis* in Invasive Rodents

After macroscopic inspection of the lung and heart tissues, we found that 46 *R. norvegicus* from five islands (the Anejima, Imoutojima, Meijima, Mukohjima, and Hirajima Islands) were infected with *Angiostrongylus* worms. The overall prevalence was 33.8% (46/136), ranging from 0% to 100% at the individual collection sites. Detailed information on the location, host species, host number, and the infection rates are presented in Table 1.

### Molecular Identification

Partial regions of the SSU rRNA gene sequence (805 bp) were separately determined for each individual worm from the Anejima, Imoutojima, Meijima, Mukohjima, and Hirajima Islands. Sequence alignment showed no variation in these sequences. Upon querying the DDBJ/EMBL/GenBank database, we found no difference between the present sequences and *A. cantonensis* (GenBank accession nos. **AB683977** [9] and **GQ181114** [20]). Based on these data, the nematodes isolated from the Anejima, Imoutojima, Meijima, Mukohjima, and Hirajima Islands were identified as *A. cantonensis.*

The partial *coxI* sequences of 46 *A. cantonensis* isolates from the Ogasawara archipelago and 2 isolates from Hawaii Islands were determined. The alignment of the *coxI* sequences of 48 isolates was 565 bp long, with no variable sites. A BLAST search against sequence databases revealed that the present sequence had 100% identity with the ac5 haplotype reported from Chichijima (GenBank accession no. **AB684372** [20]) and Hahajima (GenBank accession no. **AB68437**1 [20]) Islands in the Ogasawara archipelago, Okinawa Island (GenBank accession nos. **AB684369**, **AB684370** [20], and **AB723723**), and Brazil (GenBank accession nos. **JX471060** and **JX471063** [22]). The *coxI* sequences data reported are available in the DDBJ/EMBL/GenBank databases as follows: the Anejima (GenBank accession no. **AB700676**), Imoutojima (GenBank accession no. **AB700677**), Meijima (GenBank accession no. **AB700678**), Mukohjima (GenBank accession no. **AB700679**), Hirajima (GenBank accession no. **AB700680**), and Hawaii islands (Alicata strain) (GenBank accession no. **AB700681**).

## Discussion

### Prevalence of *A. cantonensis* in Rats

In the present study, prevalence of *A. cantonensis* in rats in the endemic islands was high, ranging from 60% to 100%. This was similar to the other endemic regions such as Cuba (60%) [27], Dominican Republic (100%) [28], Thailand (77%–100%) [29], and Fiji (60%) [30]. These findings suggested that *A. cantonensis* has already been established and is flourishing in the Hahajima group. In this study, the infection rate of *R. norvegicus* (80.7%; 46 of 57) was significantly higher than that of *R. rattus* (0%; 0 of 79), which is similar to the situations in Haiti [31] and Puerto Rico [32]. On the other hand, *A. cantonensis* worms were not detected from the rats collected in the Nakoudojima, Yomejima, Minamijima, and Iwojima Islands. These results indicate that presently, there is limited or no colonization and establishment of *A. cantonensis* on these islands.

### Historic and Current Distribution of *A. cantonensis* and Human Angiostrongyliasis in the Ogasawara Islands

In 1968, Sasa *et al*. (1969) [14] made the first survey of the Chichijima Island and discovered *A. cantonensis* larvae from *Achatina fulica* (positive ratio was 30%; 6 of 20 snails). Later surveys in the Chichijima Island confirmed widespread infections in *A. fulica* and *R. rattus* [10], [11], [12], [13], [15]. Subsequent investigations revealed the presence of infected *A. fulica, R. norvegicus,* and *R. rattus* in Hahajima [15], [16], [17] and infected *R. rattus* in the Anijima Islands [18]. Based on these reports, we surveyed the Mukojima Island (the biggest island in the Mukojima group) in 2007. However, we failed to find any infected *R. rattus* among 62 sampled individuals [18]. The other islands in the Ogasawara Islands remain uninvestigated. We then carried out a second survey between 2010 and 2011, and successfully found new endemic foci of *A. cantonensis* in *R. norvegicus*. This is the first report of the occurrence of *A. cantonensis* in the Anejima, Imoutojima, Meijima, Mukohjima, and Hirajima Islands–small satellite islands of the Hahajima group, located about 3.6–5.5 km away from the Hahajima Island. These newly detected foci could be of importance from a public health view. As far as we know, at least 67 parasitologically diagnosed human cases were reported in Japan at the end of 2007. These human cases were mostly from Okinawa Islands. Fortunately, no human cases have been reported so far in the Ogasawara Islands [11], [12], [13]. Since no clinical survey of residents in the Ogasawara Islands has been carried out since the mid 1980s, it is crucial to carry out medical examination for angiostrongyliasis in these regions.

### Phylogenetic Relationships of *A. cantonensis*

In the present study, we have provided molecular evidence that *A. cantonensis* isolated from the Chichijima group (Chichijima Island) and the Hahajima group (Hahajima, Anejima, Imoutojima, Meijima, Mukohjima, and Hirajima Islands) in the Ogasawara archipelago share a single *coxI* sequence, named haplotype ac5. This could be considered the result of a single genomic origin followed by range expansion. Furthermore, our analyses demonstrated that this shared lineage is identical in not only the Ogasawara archipelago, but also the Okinawa Islands (Japan), Hawaii (USA), and Brazil. These findings indicate that a lineage-specific *A. cantonensis* has been spreading across the Pacific.

### Current Distribution of Rodent Hosts and the Association with Introduction of *A. cantonensis*

Previous studies have shown that the 3 species of invasive rodents, namely, *R. rattus, R. norvegicus,* and *Mus musculus* (house mouse) inhabit the Ogasawara Islands. Of these species, *R. rattus* and *R. norvegicus* are capable of transmitting *A. cantonensis*. The presence of *R. rattus* and *R. norvegicus* in the Chichijima Island has been recorded since the 1920s and 1862, respectively [18], [33]. Subsequent investigations revealed the presence of *R. rattus* in virtually all islands in the Ogasawara Islands [34], while *R. norvegicus* was found only in the Hahajima, Hirajima, Chichijima, and Iwojima Islands [34], [35]. In the present report, we describe the first occurrence of *R. norvegicus* in Anejima, Imoutojima, Meijima, and Mukohjima Islands. Introduction of these rats to the Ogasawara Islands is attributed to shipping activity [34]. Even today, the ocean route connecting the Chichijima Island and Tokyo (mainland Japan) is the only way to visit the Ogasawara Islands. To verify our hypothesis–that the *A. cantonensis* population in the Ogasawara Islands originated from mainland Japan–we compared the lineages of *A. cantonensis*. Unfortunately, we could not find any shared lineages among the regions of interest and mainland Japan [20]. The dispersal of rats within the Ogasawara Islands has not yet been clearly understood. We assumed two possibilities; accidental introduction via human-related transportation and swimming abilities of rats. Previous studies have shown that *R. norvegicus* are capable of swimming hundreds of meters across open water [36], suggesting that a transmission event might have occurred.

### Epidemiology of *A. cantonensis* among Gastropods

Virtually, all gastropods act as intermediate hosts, and are capable of transmitting this parasite. In the Ogasawara Islands, the giant African snail *A. fulica* has been shown to be one of the most important intermediate hosts of *A. cantonensis* [14], [15]. It is also suggested that the migration of *A. cantonensis* in the Ogasawara Islands is responsible for the introduction of *A. fulica*. According to Mead (1961) [37] and Tomiyama (1988) [38], *A. fulica* snails were introduced to the Chichijima Island from Taiwan via mainland Japan in 1935, as an item of primitive medicine. Subsequently, the giant snails were found one after another in the Hahajima, Anijima, and Higashijima Islands. In the present study, we discovered *A. cantonensis* worms in the Anejima, Imoutojima, Meijima, and Mukohjima Islands of the Hahajima group, where *A. fulica* snails had not been reported. Therefore, we suspected that organisms other than *A. fulica* snails might be involved in the lifecycle of *A. cantonensis* in these islands. The Ogasawara Islands is abundant with terrestrial gastropods. At least 97 snail species have been recorded, and over 90% of them are native to this Islands [39]. Uchikawa (1987) [40] pointed out that rats prefer small native snails rather than large-sized *A. fulica* snails, and these small snails play a more important role in the transmission of *A. cantonensis.* However, the susceptibility of native snails in the Ogasawara Islands to *A. cantonensis* and the epidemiology of *A. cantonensis* among these snails are uncertain.

In conclusion, we demonstrated, for the first time, the occurrence of *A. cantonensis* in *R. norvegicus* from the Anejima, Imoutojima, Meijima, Mukohjima, and Hirajima Islands of the Ogasawara archipelago. We have also provided molecular evidence that a single shared lineage of *A. cantonensis* has been spreading in the Ogasawara archipelago. This lineage was found in the Okinawa, Hawaii, and Brazil. Future studied should focus on the transmission dynamics of this nematode parasite, and explore the potential role of the host in moving the parasite across a wide range of geographic regions.
